# Born to resist: Understanding natural resistance to *Haemonchus contortus* in sheep

**DOI:** 10.1371/journal.ppat.1014658

**Published:** 2026-10-05

**Authors:** Hannah Teddleton, Elizabeth Shepherd

**Affiliations:** Department of Animal Science, University of Tennessee, Knoxville, Tennessee, United States of America; University of Wisconsin Medical School, UNITED STATES OF AMERICA

## Fact 1: Infection with *Haemonchus contortus* is a major cause of clinical and economic losses in sheep driving anthelmintic resistance profiles

The hematophagous gastrointestinal nematode *Haemonchus contortus* (*H. contortus*) is widely recognized as one of the most significant parasites affecting sheep and goats, with annual losses estimated at $30–300 million globally [1]. *H. contortus* follows a direct life cycle, characterized by fecal oral transmission and a pre-patent period of 17–21 days (Fig 1). Adult *H. contortus* worms feed on blood by piercing the abomasal mucosa in the host, resulting in severe pathologies including hypoproteinemia, submandibular edema, anemia, and in severe cases, death [2]. Pathophysiology attributed to infection is characterized by anemia, caused by rapid blood loss from high parasite load, detectable ~10–12 days of infection [3]. Each adult *H. contortus* worm can consume about 50 μl of blood per day, leading to cumulative losses of 50–600 mL per day [1]. Without treatment, young or susceptible animals are vulnerable to infection, leading to nutrient imbalances, weight loss, and decreased wool and milk production [3]. Current available treatment involves the use of anthelmintic drugs, which include three major classes: benzimidazoles, cholinergic agonists, and macrocyclic lactones [2]. However, widespread use of anthelmintics has led to the development of multidrug resistance, resulting from a range of factors including genetic modifications in *H. contortus* populations, which have resulted in alterations of effective drug targets, physiochemical interactions, and drug distribution [4]. The prolific nature of *H. contortus* coupled with limited available control strategies highlights the urgent need to develop improved treatments for the effective control of *H. contortus* in sheep.

**Fig 1:**
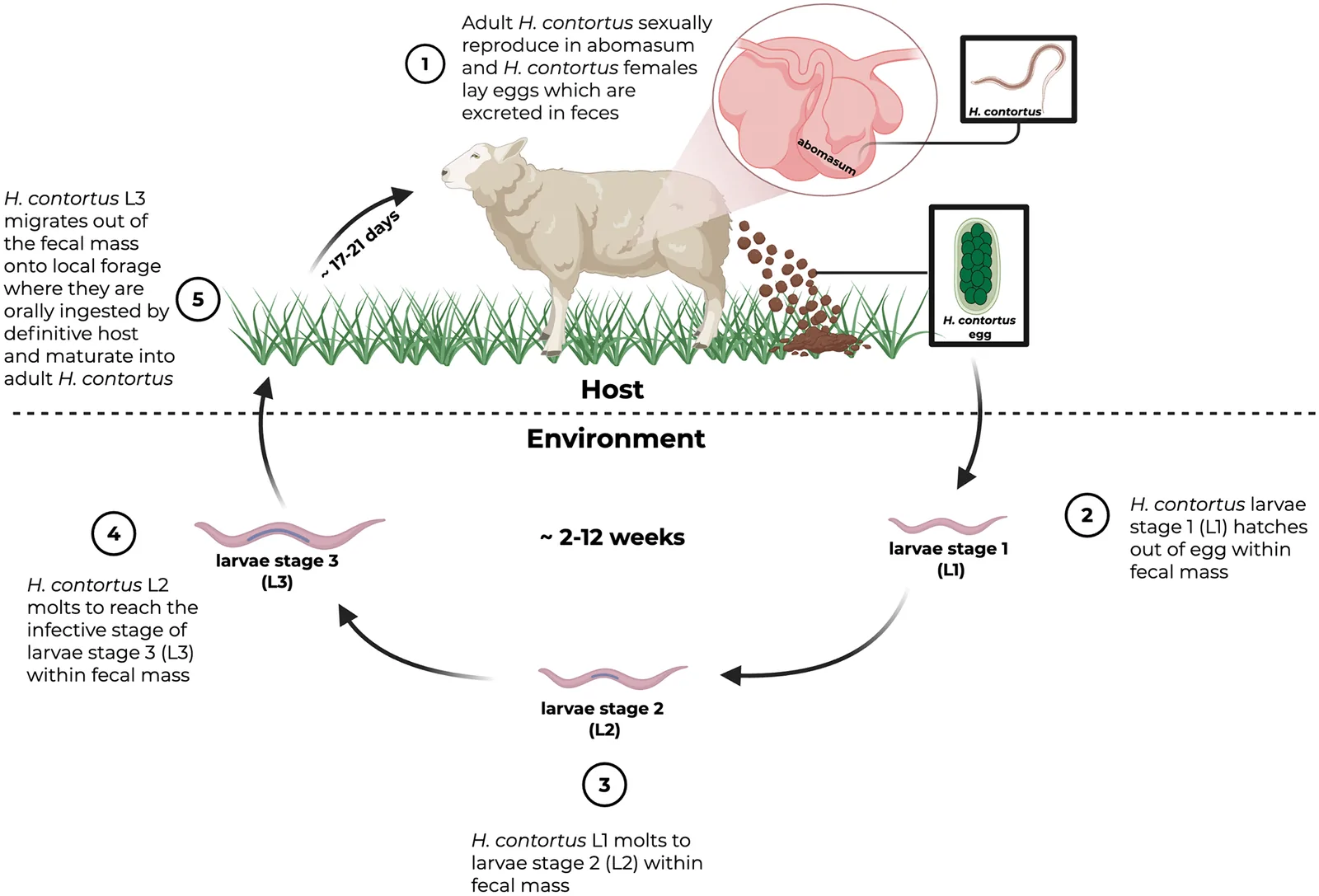
*H*. *contortus* life cycle in small ruminants. *H. contortus* follows a direct fecal-oral life cycle in small ruminants following ingestion from grazing. Created with BioRender.com/d97np7e.

## Fact 2: Different breeds of sheep display natural resistance to *H. contortus*

Sheep were one of the first domesticated animals, and although sheep share a common ancestor, substantial genetic diversity between sheep breeds influences economically relevant production traits such as carcass and wool characteristics, as well as disease resistance [5]. Natural resistance against *H. contortus* has been documented in sheep breeds, like hair breeds, which originate from humid tropical climates and have developed parasite resistance against nematodes such as *H. contortus* [6]. By contrast, wool breeds originating from the cooler environment of Europe have been selected for economically valuable traits such as carcass and wool characteristics without the environmental selective pressure against parasitism, which has resulted in increased levels of susceptibility to parasitic infection [7]. Within hair sheep, breeds such as the St. Croix, Gulf Coast Native, and Barbados Blackbelly demonstrate higher levels of resistance to *H. contortus* demonstrated by increased packed cell volumes and decreased fecal eggs counts and worm burdens in the abomasum at necropsy when compared European breeds of wool sheep such as the Suffolk and Dorset [8]. Studies investigating the resistance profiles between resistant tropical breeds demonstrated that the St. Croix demonstrated the highest resistance to *H. contortus* when compared to Gulf Coast Native, Barbados Blackbelly, and crossbred wool sheep [9], making the St. Croix sheep an ideal model for studying natural parasite resistance. However, despite their robust resistance against *H. contortus*, St. Croix sheep lack economically desirable traits, highlighting the importance of elucidating mechanisms driving a resistant phenotype in sheep.

## Fact 3: Genetic selection of sheep to be naturally parasite resistant is highly variable

While parasite resistance is influenced by factors such as sex, age, and previous exposure, host genetics are a major contributor to variation in resistance against *H. contortus* [10]. Evidence for a genetic basis of parasite resistance is supported by marked differences in resistance observed between both sheep breeds and among individuals within the same breed [10]. Therefore, identifying genetic determinants contributing to differences in parasite resistance among sheep is essential in combating *H. contortus*. Originally bred in the 1950s, Katahdin sheep are a cross between parasite-resistant St. Croix hair sheep and breeds selected for desirable production traits, including Suffolk and Wiltshire Horn [11]. Despite being widely regarded as parasite resistant, considerable variation exists in post-weaning fecal egg count (PFEC) estimated breeding values (EBV) among Katahdins, highlighting opportunities for continued genetic improvement [12]. While PFEC is moderately heritable (0.23-0.46) [11], parasite resistance remains a complex trait influenced by many genes rather than a single major genetic locus [11]. No single resistance gene has been identified, as genome-wide association studies (GWAS) and regional heritability mapping (RHM) in sheep have identified multiple regions [13], suggesting a resistant phenotype is polygenic. Consequently, understanding how these genetic differences influence immune function remains an important area of research.

## Fact 4: Resistance to *H. contortus* is mediated by immune responses

Natural resistance to *H. contortus* infection is directed against the L3 stage, preventing establishment of adult worms in the abomasum without the reliance of anthelmintic drugs [14]. In general, gastrointestinal nematodes elicit a T-helper 2 type (Th2) response in the host which is characterized by the production of key cytokines such as interleukin-4 (IL-4), IL-5, and IL-13, mediating host protection [14]. Resistance in St. Croix sheep is characterized by an early Th2 response to the abomasum (Fig 2), exhibiting increased levels of eosinophilia, mastocytosis, IgG, IgA, and production of IL-4, IL-5, and IL-13 [15]. Additionally, depletion of CD4^+^ T cells in resistant sheep resulted in increased fecal egg counts and worm burdens [16], further demonstrating that natural resistance against *H. contortus* is predicated on the development of appropriate adaptive responses. Response to *H. contortus* in susceptible breeds, like Suffolk, is delayed, permitting *H. contortus* establishment and requiring anthelmintic treatment to prevent mortality [17]. Thus, differences in the host immune response to *H. contortus* could be the mediating factor driving the resistant or susceptible phenotype in sheep breeds [17]. Despite the identification of robust Th2-like responses in resistant sheep, the exact mechanisms that drive the resistant phenotype in St. Croix remains unknown.

**Fig 2:**
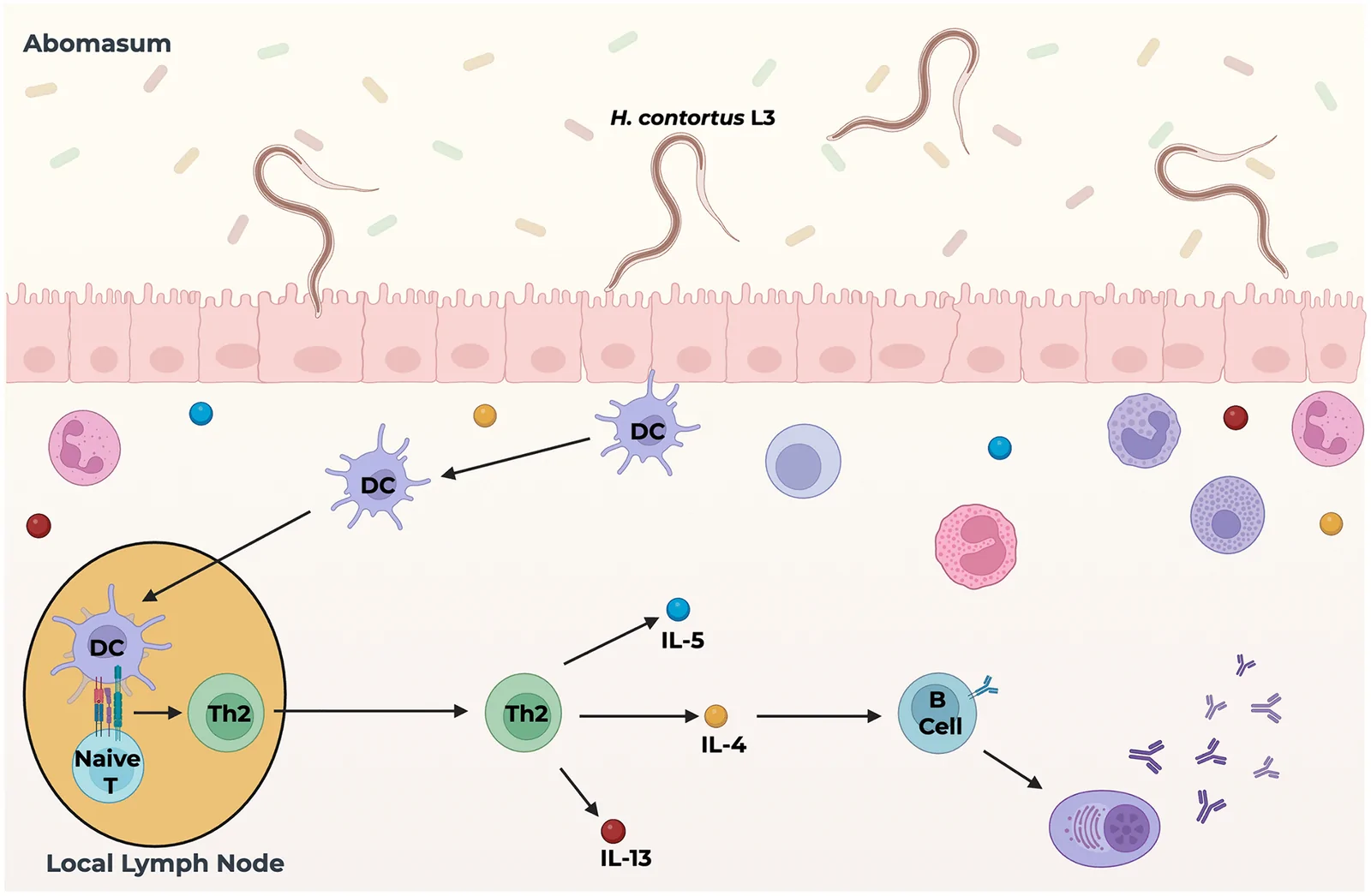
Abomasal Th2 immune response to *H. contortus* infection. Following the arrival of *H. contortus* larvae to the abomasum, the activation of innate immune cells responds to produce cytokines such as IL-4, IL-5, and IL-13 to support the development of the T-helper 2 type (Th2) polarization. Antigen-presenting cells such as dendritic cells leave the abomasum and travel to local lymph nodes such as the abomasal lymph nodes to prime naïve CD4^+^ T cells for subsequent activation and differentiation. The naïve CD4^+^ T cells differentiate into Th2 cells where they travel to the site of infection to amplify cellular responses through the production of Th2-like cytokines and to activate B cell class switching and IgE and IgA production through IL-4 production, facilitating worm expulsion. Created with BioRender.com/l2pretl.

## Fact 5: Utilization of parasite resistant sheep is a strong model to elucidate *H. contortus* mediated immunity

Apart from anthelmintic use, alternative methods of control such as the introduction of copper oxide wire particles [18] have gained popularity in the recent decade; however, application of the copper particles demonstrated relatively short anthelmintic activities in infected lambs (up to 35 days) [19] and often still requires anthelmintic drugs. While Barbervax, the only commercially available vaccine for *H. contortus*, is approved in Australia, the United Kingdom, and South Africa, the varying efficacy and high economic cost highlight the difficulties in vaccine development [20]. A protective efficacy study of Barbervax in parasite-susceptible Merino sheep showed a 60–75% decrease in fecal egg counts in low (300 *H. contortus* L3/weeks) and moderate (600 *H. contortus* L3/week) trickle infections but did not confer protection in animals given high (1,200 *H. contortus* L3/week) (2,400 *H. contortus* L3/week) trickle infections [20]. Because vaccination strategies like Barbervax may not confer protection to animals with high *H. contortus* infections [20] or high pasture contamination, alternative strategies are needed. Utilization of parasite-resistant St. Croix sheep is an ideal models to elucidate differences in the amplification of the early Th2-like response, thereby allowing for the characterization of the initiators and mechanisms that drive a resistant phenotype during *H. contortus* infection. Greater elucidation of the Th2-type immune response in parasite-resistant sheep will aid in development of cellular and cytokine-based immunotherapy strategies to aid in the control of *H. contortus* without the reliance of anthelminthic drugs.
